# The Hygiene of Motherhood

**Published:** 1891-01

**Authors:** John Sheppman


### THE HYGIENE OF MOTHERHOOD.

BY DR. JOHN SHEPPMAN.

PART THIRD.

The next subject of vital importance in connection with these series is the proper care of the home, and how much sickness can be prevented by a knowledge of the prohibitory measures to be taken in cases of contagious diseases. Too much emphasis cannot be laid upon the necessity of the co-operation of the women in all matters advanced by physicians for the progress of sanitary science. Every one knows that the female sex is particularly fitted to administer to the wants of the weak and the afflicted ; and no one will question the admirable consideration of nature in creating women with a special faculty for the performance of our household duties. Now, admitting the natural qualifications with which we are individually endowed, it certainly becomes manifest that we are all expected to labor in our given pursuits, and by labor we not only benefit the general conditions of mankind, but build up, strengthen and fortify ourselves against the bitter conflicts which unexpectedly assail us in our various walks of life.

The higher order of intelligence, the executive and constructive abilities are doubtless to be found in man. To his skill we must credit the phenomenal growth of our cities, the marvelous discoveries of science, the ingenious mechanical improvements, and the general impetus which is given to all branches of studies which have a moral, physical, and elevating influence upon all classes of humanity. The creation of woman came second, in providential wisdom. Not only does she exist for the continuance of the human race, but as a helpmeet for man ; to love and console, to soothe and comfort, to cheer and respect him in his tedious efforts to maintain a respectable home, and to live with a serene contentment through an undeviated life of wedded bliss. The unique completeness and vestal relation of the sexes can only be found where husband and wife are joined together not merely for the gratification of their sensual pleasures, but with a delight in each other's presence, a love of the soul, and the heart, that pours back its affections with the ardent breath of sincerity, that looks forward to a higher social development, and a nobler effort to sustain all things that are fair, clean, and purely righteous. True domestic felicity can always be seen where men and women work with one congenial motive, and where the woman makes assiduous efforts to keep her home cheerful.

One of the principal causes for the prevalence of bachelorhood can be traced to the utter indifference or the carelessness which our marriageable young ladies show towards all household subjects. Every intelligent man who contemplates matrimony will question the capabilities of his affianced in this respect, and no doubt the neglect, or lack of this particular knowledge has had much to do with framing many of the unpleasant suits for "breach of promise." The highest degree of civilization is shown by the union of the sexes, and when this becomes impracticable, then mankind sinks beneath the level of the brute.

I do not wish my lady readers to be misled by these statements, nor to think that I am trying to cast a damper on female energies ; such is not my purpose. If a woman's ambition soars in manly pursuits, and she meets with success, let her continue her labors, for, after all, the chief inherited trait of our existence seems to be an accumulative thirst for wealth, and why should not the female sex be granted an equal privilege in this pecuniary strife. Of course there are exceptional conditions which we all must acknowledge, but no woman should permit herself to fall into the habits of man, and to forget the designated duties which nature has set apart for her. Although you may be much superior to your husband, in wisdom, and learning, it is best always to consult him in matters of business, for these little inquiries lead to a greater confidence, strengthen dignity, and make him feel and know that *he* is the man, and *you* are the woman.

The relation of hygiene to the proper care of the home is one of the nearest kin, for no one can be capable of managing a house without some knowledge of sanitary precautions. The popular mind has been too much abused by elaborate detailed essays filled with technicalities on the detrimental influences of sewer gases ; and the word-science in connection with this study, has no doubt had a sombre, scaring effect upon many females. A brief review of the general information which ought to be disseminated among all young women must therefore be appropriate.

We will first direct our steps to the lowest apartment of our residences—the cellar. Much has already been said in reference to its cleanliness, and how much respect we owe to this necessary portion of our households. Many women are in the habit of placing all kinds of rubbish in this apartment, little thinking that most of the air which they breathe in their upper chambers has first passed through the cellar, and been vitiated by all foul and decaying substances. In order to convince those who are sceptical of the truthfulness of this assertion, we need only to advise them to close all upper room doors, and to boil onions in their cellars. The odor of this vegetable will be perceptible from parlor to attic, and proves beyond question that much sickness is propagated in this manner. Dryness and ventilation are particularly necessary, as many cases of fever and diphtheria can be traced to this neglect. Fruits and vegetables, and indeed, every article of corrosive properties, are more apt to decay if left in a place where the surroundings are damp. The absolute necessity of a clean, dry cellar requires us to whitewash all walls and ceilings at least twice a year, and to place some lime and charcoal in different receptacles as often as the feeling of dampness can be experienced. These articles are recommended to purify the air, as well as to absorb moisture.

Little need be said about the kitchen, for every one knows that the principal instructions would be embraced in this one word—cleanliness. It is in this room that the habits of a woman can best be discovered, and where her filthy faults are manifestly proven. There is nothing more disgusting than the presence of vermin, and their existence depends chiefly upon the slothful habits of the cook. Powdered borax has been found very efficient in removing that predominant kitchen pest—the cockroach. Other expedients might be suggested, but the best of all can be found in the virtue of good common soap, with plenty of hot water.

The dining room should always be regarded as a place of pleasure, where families gather, not merely for the purpose of satiating their appetites, but to indulge in a free and pleasant discourse on the sociable topics of the day. All business cares should be discarded when entering this apartment, and a feeling of blissful serenity should promptly take possession of our busy minds. It is this control that allows us to feel the cravings of hunger, that assists the process of digestion, and that aids immensely in correcting the evils which ultimately lead to a chronic state of dyspepsia. Bright pictures, very often, have a charming influence in this respect.

It is a difficult matter to describe what care should be taken of the parlor, for each individual has a special taste of furnishing and decorating, which seems to be the entertaining subject among many women. This room, although little occupied, requires ventilating every morning, and the shutters should be allowed to remain open through the entire day. The same advice may be applied to the bed-room, or chamber, with a little more emphasis, and an earnest plea for plenty of fresh air, and a greater welcome extended to the purifying rays of the ascending sun. It is an unhealthy practice for elderly people to sleep in the same bed with children, and as few as possible should be confined in the one room. No fire, heat, nor light of any kind should be burnt all night, for they impoverish the air, and unquestionably "feed upon our very vitals." Cold, damp beds are extremely dangerous, and no one should enter the chilling sheets without first having wrapped themselves securely in a good woolen blanket. The housemaid should never forget to turn the mattresses in the morning, and to thoroughly air the sheets, quilts and pillows. Water that has stood in a bedroom over night is not considered pure, and must therefore be used for washing purposes only. The spare room should be cleaned and heated at least once a week, to prevent the settling of a killing dampness.

If you move to the country in the summer, always make it a point to visit your city house one day out of the seven, and let it be your duty to open all windows and to turn on all water flows, for the plumbers' modern safety "traps" become useless and allow the sewer germs to take full possession of your homes, if the water in them has been permitted to evaporate.

There are many other suggestions which I desire to make, but as I have already occupied so much space, I will reserve them as the subject for my next part.

When the women learn to apply themselves to the study of the art of making their homes cheerful, then, and not until then, will the club rooms, grog shops and poker dens lose their patronage ; the men will be more devoted to their wives, and will look forward, at the close of day, with blissful anticipation to the joys to be found with their own families, at the side of the hearth, where mothers and children sit with hallowed grace, the true and living pictures of that dear old melody—"Home, Sweet Home."

#### PLAGIARISM AND THE PROOF-READER.

In part second of "The Hygiene of Motherhood," our proof-reader neglected to notice the little quotation marks which our extremely sensitive contributor had placed before and after the lines "Health must be there or beauty cannot be," etc. In order to acquit him of any charge of plagiarism, we have consented to insert this apology.

